# Puberty and Sex Education Challenges in Adolescents With Autism Spectrum Disorder: Mixed-Methods Evidence From Lebanon

**DOI:** 10.7759/cureus.102518

**Published:** 2026-01-28

**Authors:** Issa Almoussawi, Hiba Barakat, Zaynab Ghrayeb, Fatima Dagher, Hussein M Ziab

**Affiliations:** 1 Special Education, University of Sciences and Arts in Lebanon, Beirut, LBN; 2 Al-Maha Pediatric Specialized Care Center, Hamad Medical Corporation, Doha, QAT

**Keywords:** adolescents, autism spectrum disorder, cultural barriers, puberty, sex education, teacher training

## Abstract

Background

Autism spectrum disorder (ASD) is a complex neurodevelopmental condition marked by deficits in social communication and behavior. Adolescents with ASD experience puberty similarly to neurotypical peers but face amplified challenges due to impaired emotional regulation and social cognition. In Lebanon, the absence of formal sex education and prevalent cultural taboos compound these difficulties.

Objective

This study explores puberty and sex education experiences among Lebanese adolescents with ASD, emphasizing intervention needs by gender and ASD severity from the perspectives of parents, educators, and allied professionals.

Materials and methods

Using an exploratory sequential mixed-methods design, Phase 1 comprised semi-structured interviews with nine parents and three allied professionals. Phase 2 involved a quantitative survey of 36 special education teachers. Qualitative data underwent thematic analysis, while quantitative responses were examined using descriptive statistics and correlation analyses.

Results

Adolescents with ASD face heightened emotional, sensory, and behavioral difficulties during puberty, including boundary confusion and hygiene challenges. Severity and gender influenced intervention needs: females faced more sensory and hygiene burdens, while severe ASD cases required individualized strategies. Teachers reported moderate resource access, high discomfort with sex education, and strong demand for training and curriculum reform. Embarrassment was negatively correlated with perceived teaching capability (r = -0.38, p = 0.021); parental cooperation and resource access were positively associated (r = 0.54 and 0.69, respectively).

Conclusions

There is a pressing need for culturally sensitive, gender-specific sex education programs for adolescents with ASD in Lebanon. Findings support policy reform and tailored interventions in conservative or resource-limited contexts.

## Introduction

According to the American Psychiatric Association (2022), autism spectrum disorder (ASD) is considered a complex and lifelong neurodevelopmental disorder specified by limited and repetitive patterns of behavior, interests, or activities in addition to ongoing deficiencies in social communication and interaction [1]. Individuals with ASD may have difficulty modifying their behavior to fit different social situations, especially in recognizing nonverbal cues, interpreting facial expressions, and initiating conversations [2]. Significant gaps remain in the development of therapeutic and educational interventions tailored to the changing requirements of people with ASD, especially during adolescence [3,4], even though the prevalence of ASD has dramatically increased globally in recent years, with estimates putting it currently at 1 in 36 children [5]. Puberty is defined as the biological process leading to sexual maturity, characterized by physical changes such as menstruation, the onset of ejaculation, hormonal shifts, the development of secondary sexual characteristics, and genital growth [6]. Puberty typically occurs during early adolescence, while adolescence is a broader developmental stage spanning the transition from childhood to adulthood. During adolescence, individuals experience not only biological maturation but also major psychological and social changes, requiring increased emotional regulation and social awareness to manage emotional variability, heightened social pressures, and evolving identity roles [7].Adolescents with ASD, as well as neurotypical individuals, face difficulties in such transitions. Still, they are much more difficult for those with limited social cognition, heightened sensory sensitivity, and impaired cognitive flexibility [8].

Sexual development is not excluded for people with ASD; while 75% of adolescents with ASD engage in sexual conduct during adolescence, they frequently lack awareness of social standards [5]. Adolescents with ASD also experience sexual feelings and actions; meanwhile, because of a misinterpretation of social cues, about 30% of these behaviors, which may include public masturbation and boundary violation, are inappropriate from a social view [2]. These actions are explained as resulting from deficiencies in Theory of Mind (ToM), a cognitive ability that allows one to infer the intentions, feelings, and ideas of others [9]. Deficiencies in ToM make people with ASD more likely to encounter social exclusion or poor judgment because they are unable to estimate how their actions will affect others, especially in private situations.

Inclusive sex education is receiving attention worldwide, but cultural taboos around gender roles, menstruation, and sexuality can still limit institutional planning as well as parental communication [10]. Youth with disabilities encounter additional obstacles in Lebanon, where sex education is completely excluded from official curricula, and families frequently deal with puberty-related changes without the assistance of qualified specialists or culturally appropriate resources [11,12].

The puberty experience of teenagers with ASD appears to show gender-specific patterns. Female adolescents with ASD report poorer levels of social and medical support and are more likely to experience emotional dysregulation and sensory discomfort during their periods [13]. However, male adolescents with ASD have some abstract cognitive understanding of sexual boundaries. As a result of poor emotional regulation, they may exhibit more externalizing behaviors, such as inappropriate touching or sexualized language [14].

The severity of ASD symptoms is another important consideration. While adolescents with severe or non-verbal autism need more customized, behaviorally grounded, and visually based treatments, adolescents with mild-to-moderate ASD may benefit from organized educational interventions [3,15]. For teaching socially acceptable behavior and self-care skills, interventions such as visual timetables, social storytelling, and applied behavior analysis are particularly effective [12]. However, few schools in Lebanon offer customized curricula, and many teachers lack the institutional support and training necessary to apply these strategies [16].

These difficulties are made more difficult by the pervasive parental worry that sex education would promote sexual behavior. However, there is no empirical data to support this assumption. Conversely, studies show that among adolescents with ASD, developmentally appropriate, culturally sensitive, and visually sensitive sex education improves hygiene, lowers inappropriate behavior, and reduces anxiety [17].

In conclusion, there is an urgent need for focused educational initiatives to serve adolescents with ASD during puberty due to the junction of neurological impairment, cultural taboo, and systemic gaps. This study aims to investigate the difficulties encountered by teenagers with ASD in Lebanon from the viewpoints of both parents and educational experts. With consideration for gender, the degree of autism, and sociocultural limitations, the study specifically looks at the lived experiences and methods used by professionals and caregivers to deal with puberty and sex education. The ultimate goal is to produce evidence-based, context-specific recommendations for inclusive, well-organized educational interventions.

## Materials and methods

Study design

Given the sensitive, under-researched nature of the topic, an exploratory sequential mixed-methods design was used. This design involved an initial qualitative phase followed by a quantitative phase, with insights from the first informing the second. The rationale for choosing an exploratory sequential approach over a convergent design was twofold. First, qualitative interviews provided in-depth context and uncovered emergent themes in the Lebanese ASD community; these findings were used to shape the content of the subsequent teacher survey instrument. This ensured that the quantitative measures accurately reflected the issues and terminology arising from participants' personal experiences. Second, given the lack of existing instruments or prior data in this cultural context, the sequential design maximizes relevance and cultural sensitivity. In contrast, a convergent parallel design (conducting qualitative and quantitative components simultaneously) would have required a predefined questionnaire that might not capture crucial context-specific aspects. The exploratory sequential design ultimately balanced the richness of contextual insights with the empirical breadth of a survey, providing a comprehensive understanding of puberty and sex education challenges among adolescents with ASD in Lebanon.

The scientific committee of the University of Sciences and Arts in Lebanon approved the study’s protocol prior to data collection. Moreover, this project was approved by the Institutional Review Board of the University of Sciences and Arts in Lebanon (approval number: USALIRB-2025-1). All participants signed a written consent form of their agreement to participate in this study.

Participants

**Figure 1:**
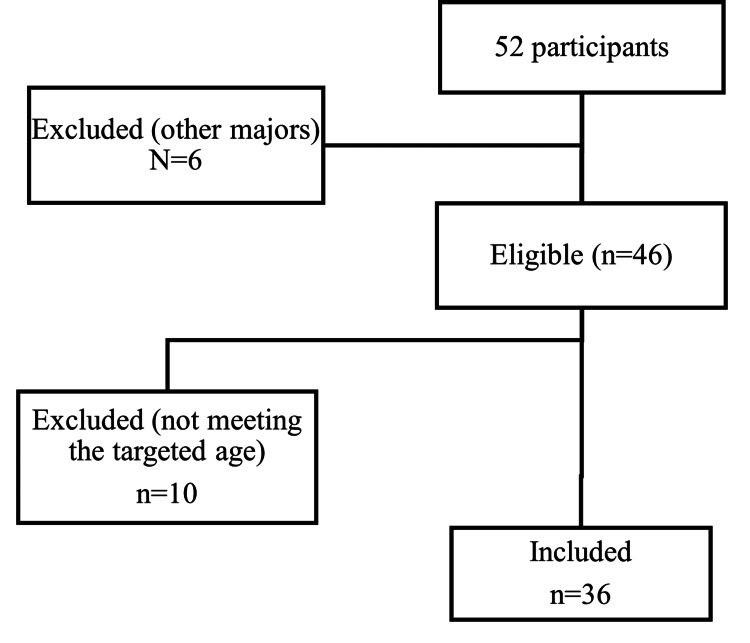
CONSORT flowchart for the special education teachers Major refers to the participant's specialty; six of them did not hold a degree in special education (some were assistant or regular teachers working in the center).

The study involved 48 participants, divided into three groups. Special education teachers (n = 36) completed the quantitative survey. These teachers were all certified professionals working with adolescents with ASD (aged 10-19) in Lebanon, recruited through an online snowball sampling strategy. Initial outreach via professional networks and social media yielded 52 responses; after excluding ineligible respondents (e.g., those not working in the target age range or without the relevant specialization), 36 valid teacher questionnaires remained. The teacher sample was predominantly female (reflecting the field’s demographics) and spanned a range of early- to mid-career educators. Most held degrees in special education or related fields, with varying years of experience (2-15 years) in teaching adolescents with ASD. Although broad geographic data were not explicitly collected, respondents came from multiple schools and centers across Lebanon, providing a diverse cross-section of special education settings (Figure 1).

Parents (n = 9) of adolescents with ASD participated in the qualitative phase through semi-structured interviews. Eight mothers and one father, each having a child with ASD aged 10-19, were recruited purposively via autism support networks and special schools (Table 1). Recruitment continued until thematic redundancy was reached, and saturation was judged to occur when no new themes emerged in successive interviews. All parent participants lived in Lebanon and were directly involved in their child’s care and educational planning. Given the relatively homogeneous context (parents dealing with ASD puberty challenges in one cultural setting), this sample size was considered sufficient for capturing the major themes.

**Table 1:** Parents their children demographics ASD: autism spectrum disorder

Participant	Relationship	Adolescent age (years)	Gender	ASD severity	Communication	Medication
Parent 1	Mother	12	Female	Severe	Verbal, few words	No
Parent 2	Mother	15	Male	High-functioning	Verbal	No
Parent 3	Mother	13	Female	High-functioning	Verbal	No
Parent 4	Mother	14	Female	Moderate	Non-verbal	Yes (risperdal)
Parent 5	Mother	13	Male	Moderate	Verbal, few words	No
Parent 6	Mother	14	Female	Moderate	Non-verbal	No
Parent 7	Mother	12.5	Male	Severe	Non-verbal	Yes (six medications)
Parent 8	Mother	14	Male	Moderate	Verbal	No
Parent 9	Father	15	Male	Mild	Verbal	Yes (risperdal)

Allied health professionals (n = 3) were also interviewed to incorporate expert perspectives. This small group comprised a board-certified behavior analyst (BCBA), an occupational therapist (OT), and a psychiatrist, each with over 10 years of experience working with adolescents on the autism spectrum. They were selected via purposive sampling to represent distinct professional lenses: behavioral therapy, sensory-motor therapy, and medical/psychiatric care. While limited in number, these key informants provided valuable expert corroboration and nuance to complement the perspectives of parents and teachers.

Sampling limitations

The authors acknowledge certain sampling constraints. The qualitative samples (parents and professionals) were primarily recruited by convenience and network referrals; thus, they may not capture the full variability of experiences in the broader population. In particular, the allied professional sample was very small (n = 3), so findings from that group should be interpreted as illustrative rather than generalizable. Similarly, the teacher survey utilized snowball sampling, which can introduce bias and limit generalizability [18]. Participants likely had connections to the initial distribution channels, potentially over-representing educators who are primarily engaged or resourced. To mitigate this, a vast network was cast across different schools and regions in Lebanon, but the sample is not random and may underrepresent teachers from less-connected communities. These limitations are acknowledged when interpreting the results; the findings highlight patterns and associations worthy of further study rather than making definitive population estimates.

Instruments

Two primary instruments were developed for data collection, aligned with the mixed-methods design.

Special Educators Survey

A structured questionnaire was designed for special education teachers to quantitatively gauge puberty-related challenges, practices, and needs. The survey consisted of 35 items divided into five thematic sections: (A) personal and professional profile, (B) puberty-related challenges observed, (C) intervention strategies used, (D) available resources, and (E) educator preparedness and training needs (Table 2, Annex 1). Questions were formatted as dichotomous (yes/no) or Likert-type (primarily 5-point rating) scales, allowing for frequency or agreement responses. The content was informed by both existing literature and the exploratory qualitative findings. In particular, prior studies on sex education and ASD informed the initial framework of topics [5,15,19].

**Table 2:** Content structure of the special educators questionnaire

Section	Content area	Items
A	Personal and professional profile	6
B	Puberty-related challenges	7
C	Intervention strategies	11
D	Educational resources	5
E	Educator preparedness	6

Additionally, themes emerging from the early parent and professional interviews were incorporated to ensure the survey addressed culturally specific issues and language identified in the Lebanese context. The draft questionnaire underwent expert review by two specialists in special education and educational technology to establish content validity. Minor refinements were made based on their feedback. A small pilot test with a handful of teachers was then conducted to check clarity and comprehension; this led to minor wording adjustments and confirmed that the items were generally well understood. To assess internal consistency, Cronbach’s alpha was calculated for each of the questionnaire's five sections. Reliability coefficients ranged from α = 0.76 to α = 0.84 across the domains, demonstrating acceptable internal consistency.

Interview Guides

Semi-structured interview guides were developed separately for allied professionals (Annex 2) and parents (Annex 3) to shape the qualitative inquiries. These guides were informed by frameworks from previous qualitative research on adolescents with ASD and puberty [3,12]. The parent interview guide emphasized eliciting lived experiences: parents were asked to describe the puberty-related changes observed in their child, the challenges encountered (e.g., in hygiene, sexual behaviors, emotional regulation), how they responded or coped, and what resources or information would help them. The professional interview guide, while overlapping on some topics (such as behavioral issues during puberty), focused on clinical and educational practices, for example, how professionals address puberty and sexual education in their work, their strategies for teaching appropriate behaviors, and their perspectives on systemic or cultural barriers in Lebanon. Both guides used open-ended questions with probes, allowing participants to speak in depth and introduce new insights. This flexible format enabled the capture of nuanced information while maintaining enough structure to ensure key domains were covered across interviews. The guides were reviewed by the research team (and a bilingual educator) to ensure questions were clear and culturally appropriate. Interviews were conducted in Arabic, as all participants were native Arabic speakers. The interview guides were developed in Arabic to ensure comfort and clarity, and transcripts were translated into English by a bilingual researcher for analysis and reporting.

Data saturation

We continued parent recruitment until we observed thematic redundancy, defined as the point at which new interviews yielded few new codes or themes. By the ninth parent interview, no new themes emerged, suggesting that thematic saturation had been reached in this context.

Data collection

Data were collected over a five-month period (January-May 2025) using distinct approaches for the two study phases. In the quantitative phase, the teacher questionnaire was administered electronically. An online survey link (via Google Forms, Google LLC, Mountain View, CA, USA) was distributed via email, professional WhatsApp groups, and social media platforms for special educators. We relied on a snowball sampling approach; initial contacts were encouraged to forward the survey to other eligible teachers to maximize reach across Lebanon's regions. Respondents completed the survey anonymously at their convenience; submission of the form implied consent. By the end of the collection period, 36 completed teacher questionnaires met the inclusion criteria and were retained for analysis.

For the qualitative phase, in-depth interviews were conducted with parents and allied professionals. Participants were contacted directly and invited to an interview at a time and via a medium of their choice. Three professional interviews were carried out either face-to-face in a private office or via video conferencing (Zoom Communications, San Jose, CA, USA) for convenience. Parent interviews were predominantly conducted in person, with a few conducted via WhatsApp Voice Call (Meta Platforms, Inc., Menlo Park, CA, USA) when in-person meetings were not feasible. Each interview lasted approximately 30-45 minutes. In total, about four hours of interview data were collected (mean interview length ~30 minutes). With participants’ consent, all interviews were audio-recorded to ensure accuracy of data capture. The recordings were subsequently transcribed verbatim. The transcripts of the interviews were translated into English by a bilingual researcher, taking care to preserve the meaning and nuance of participants’ expressions. The interviewer cross-checked each translation against the original Arabic transcript to ensure fidelity. All identifiable information was removed or coded during transcription to maintain confidentiality.

Data analysis

Qualitative Data Analysis

The interview data were analyzed using an inductive thematic analysis approach, following the guidelines of Maguire and Delahunt (2017) [20]. Taguette software was used to facilitate data management and coding [19].First, all transcripts were read in full to achieve immersion in the content. Initial coding then proceeded line-by-line: two members of the research team independently coded each transcript, assigning labels to meaningful units of text. Recruitment continued until thematic saturation was reached, defined as the point at which no new codes or themes emerged across successive interviews. Coding was largely data-driven (no pre-set codebook), so codes reflected concepts emerging from participants’ own words. The two coders compared their coding after each transcript and discussed any discrepancies in code application or interpretation. Through iterative refinement, a consensus codebook was developed, and earlier transcripts were revisited to ensure consistent application of the finalized codes. This collaborative coding process enhanced reliability by strengthening coding consistency through independent coding and consensus discussions, with all disagreements resolved through discussion rather than a numerical inter-rater metric.

After open coding of all transcripts, related codes were clustered into candidate themes and subthemes. The research team (including members with backgrounds in special education and clinical practice) reviewed and refined these themes, ensuring each was well-supported by the raw data and distinct from the others. Five overarching themes ultimately emerged, encompassing: (1) physiological and sensory distress during puberty; (2) boundary confusion and inappropriate sexual behaviors; (3) gaps in interventions and support strategies; (4) gender- and severity-specific patterns; and (5) systemic cultural barriers and taboos. These themes captured the recurrent issues highlighted by participants and formed the organizing framework for reporting the qualitative findings.

Throughout the qualitative analysis, triangulation of sources was used to enhance trustworthiness. By comparing the perspectives of parents, teachers, and clinicians, the key findings were cross-verified to ensure that the themes reflected a convergence of evidence rather than a single group’s subjective view. Moreover, an audit trail of the analysis decisions (e.g., memos on code definitions and theme development) was maintained to support dependability. Although formal member checking was not conducted (given the one-time interviews and the sensitivity of the topic), the consistency of the themes across multiple participants and stakeholder groups suggested credibility of the results. This reflexive approach throughout the process aims to acknowledge how the professional backgrounds of the interviewers and researchers might influence interpretation. It deliberately grounds the analysis in participants’ actual quotes to mitigate bias. In reporting the qualitative results, the researchers adhered to the Consolidated Criteria for Reporting Qualitative Research32-item checklist for transparency in qualitative research methods [21]. Overall, the analysis was an iterative, rigorous process aimed at faithfully representing participants’ experiences and perceptions.

Quantitative Data Analysis

Survey responses from the 36 teachers were compiled and analyzed using SPSS Statistics version 28 (IBM Corp. Released 2021. IBM SPSS Statistics for Windows, Version 28.0. Armonk, NY: IBM Corp.). Initial steps included data cleaning and screening. As the online form required responses to all key questions, no imputation was necessary. The distribution of each quantitative variable was examined. Likert-scale items (rated 1-5) were treated as approximately interval-level, particularly when aggregated into composite scores. Key indices, such as a teacher “capability” score and a “parental cooperation” score, were calculated by averaging or summing relevant questionnaire items. These composite variables exhibited roughly symmetric distributions. Normality was assessed using Shapiro-Wilk tests and Q-Q plots, confirming that the assumptions of Pearson's correlation were met for the primary variables of interest, thereby justifying the use of parametric tests.

Bivariate analyses were conducted to examine associations between variables identified as thematically relevant from the qualitative phase. Pearson’s r correlation coefficients were calculated among variables such as teachers’ self-rated capability in delivering puberty or sex education, perceived parental cooperation, access to resources, personal discomfort or embarrassment with the topic, and advocacy for implementing a formal sex education curriculum. These variables corresponded to questionnaire items and scales that mapped onto key qualitative themes. For example, teacher capability was examined in relation to parental support and resource access, and embarrassment was explored as a potential inverse predictor of curriculum advocacy. All correlations were two-tailed. Given the exploratory nature of the study and the limited sample size, significance was set at p < 0.05 without Bonferroni correction to avoid increasing Type II error. To reduce over-testing, only a priori variable pairs were examined, and findings were interpreted cautiously. Both r-values and p-values were considered, with greater weight placed on whether observed patterns aligned with qualitative insights.

To ensure robustness, Spearman rank-order correlations were also conducted as a non-parametric sensitivity analysis, appropriate for ordinal data. The Spearman coefficients mirrored the Pearson results in direction and significance across key variables, increasing confidence that any violations of normality did not affect conclusions. Descriptive statistics (frequencies and percentages) were also calculated to describe the prevalence of observed behaviors, challenges, and resource use among educators. These results were later integrated with qualitative themes to provide a multi-dimensional interpretation. More complex inferential tests, such as regression or group comparisons, were not pursued due to the small sample size and the study’s exploratory purpose. Instead, the quantitative phase was designed to support and enrich the qualitative findings with empirical associations.

Integration of Qualitative and Quantitative Findings

Consistent with the exploratory sequential design, the qualitative and quantitative components were intentionally integrated at multiple stages of the research process. During the instrument development phase, insights from the initial qualitative interviews directly informed the construction of the teacher survey, exemplifying a “connecting” integration in which Phase 1 findings shaped the structure and content of Phase 2. Following data collection, a deliberate merged analysis was conducted at the interpretation stage to generate meta-inferences from both data strands. Themes emerging from the interviews were compared against statistical patterns in the survey data to identify points of convergence, complementarity, or divergence.

In the Results section, findings are presented by thematic domain, each incorporating both qualitative perspectives (illustrative quotes and summaries from parents and professionals) and corresponding quantitative data (survey-derived frequencies and correlations). This side-by-side structure functions as a narrative joint display, enabling the integration of evidence within each analytic domain. For example, a qualitatively identified theme of “cultural silence” surrounding sexuality was reflected quantitatively by a significant negative association between teacher embarrassment and support for curriculum reform, demonstrating consistency across data types. Overall, a high degree of convergence was observed between qualitative and quantitative findings. The survey data provided numeric validation and scope for the experiences described by participants in interviews, reinforcing the central themes without contradiction. Additionally, the integration revealed more nuanced understandings; for instance, qualitative accounts of gendered puberty challenges were quantitatively supported by prevalence data differentiating male and female student behaviors.

Throughout this integration process, best practices in mixed-methods reporting were applied to ensure transparency and credibility [22]. The combined analysis yielded a more comprehensive and robust interpretation than either method alone could achieve. Qualitative data illuminated potential explanations behind observed statistical relationships, while quantitative data clarified the extent to which certain perceptions were shared among teachers. This integrated evidence base informed the development of grounded, culturally relevant recommendations for intervention and policy.

## Results

Findings are organized into five domains: (1) puberty-related changes and challenges, (2) role of severity, (3) gender-specific considerations, (4) intervention practices, and (5) resources and preparedness. Each domain integrates qualitative insights from parents and specialists with survey results from special educators, and then presents convergence statements (Table 3).

**Table 3:** Qualitative themes with illustrative quotes BCBA: board-certified behavior analyst, OT: occupational therapist,P: parent

Theme	Specialists (BCBA/psychiatrist/OT)	Parents
Behavioral challenges	“Sexual behaviors are the worst… they don’t think about consequences” (Psychiatrist)	“He used to take off his pants even in front of strangers” (P6)
Sensory sensitivities	“Huge sensory needs limit options” (BCBA)	“He refused to shave because he became a man” (P8)
Emotional regulation	“Autism is accompanied with anxiety and depression in adulthood” (BCBA)	“She developed aggressive behaviors, hitting her siblings” (P4)
Hygiene and self-care	“Difficulty with pads, bras, hygiene” (OT)	“She can’t change the pad on her own” (P1)

Puberty-related changes and challenges

*Specialists* described overlapping but distinct concerns. The BCBA highlighted social-communication gaps (“They talk about what interests them… not what the other person is saying”), sensory sensitivities, and emotional vulnerability. The psychiatrist emphasized agitation, aggression, and sexual behaviors such as undressing or masturbation in public. The OT underscored sensory discomfort with clothing, hygiene, and shaving.

*Parents* repeated these difficulties, citing mood swings, aggression, self-injury, and lack of hygiene management. One mother explained, “She can’t change the pad on her own… she doesn’t even know it must be changed every few hours.” Another father reported, “He used to take off his pants even in front of strangers.”

*Educators* confirmed the high prevalence of such behaviors. Touching private parts (70.3%), hugging (67.6%), and masturbation (54.1%) were the most frequent concerns. Mood swings (56.8%) and reliance on teachers for contact (59.5%) were also prominent. Tactile hypersensitivity related to skin and hair changes was most common (59.5%). Emotional regulation difficulties (73%) were the leading trigger of meltdowns (Table 4).

**Table 4:** Prevalence of puberty-related behaviors reported by educators

Behavior	Reporting
Touching private parts	70.3%
Hugging	67.6%
Masturbation	54.1%
Mood swings	56.8%
Physical contact with the teacher	59.5%
Undressing in public	43.2%
Rubbing oneself with objects	43.2%
Kissing	37.8%
Pinching/lack of personal space/interest in the opposite sex	32.4%

Puberty increased emotional, behavioral, and sensory difficulties in all groups. Parents emphasized family stress, educators measured classroom frequency, and professionals focused on clinical categories. These results collectively suggest that sexual behaviors, sensory overload, and emotional dysregulation are key puberty-related issues in ASD.

Role of severity

*Allied health professionals* link severity to communication abilities. The BCBA noted, “When the language is limited, usually you see more problem behaviors?” The psychiatrist stressed that interventions are more effective with mild-to-moderate cases. At the same time, the OT suggested that high-functioning adolescents may pass puberty with fewer interventions compared to severe cases requiring intensive support. Educators supported this view: 40.5% reported that severity had a significant effect on sex-education uptake, and 24.3% rated this impact as extreme. Parents similarly described greater confusion and difficulty preparing nonverbal or minimally verbal adolescents for puberty.

Communication ability, often a proxy for severity, consistently emerged as a central factor influencing both the nature of challenges and the effectiveness of interventions. Quantitative and qualitative data aligned in demonstrating that greater severity required more individualized and intensive support strategies.

Gender-specific considerations

*Specialists* emphasized differences in both sensory and behavioral domains. The BCBA and OT noted that menstruation heightened sensory distress for girls (pads, bras, pain), while the psychiatrist stressed impulsivity and aggression in boys linked to testosterone and body size.

*Parents* confirmed these distinctions: mothers of girls described hygiene anxieties and menstruation difficulties, while parents of boys reported aggression and public masturbation. Preparation strategies also diverged; families of girls often used proactive approaches (e.g., pad training), while families of boys tended to react once challenges emerged.

*Educators* confirmed this gendered distinction, with 67.6% acknowledging that puberty challenges differed by gender; 66.7% viewed girls as less capable of coping, compared to 37% for boys.

Gender clearly moderates the type and timing of challenges. Females faced sensory and hygiene-related struggles, while males displayed more impulsive and boundary-crossing behaviors.

Intervention practices

*Specialists* advocated a proactive, multidisciplinary approach. The BCBA described social skills training, self-monitoring, discrimination training, cyber safety, vocational training, and physical activity. The psychiatrist stressed therapy and, in severe cases, medication to manage aggression or sexual behaviors. The OT emphasized sensory-based preparation (e.g., training girls on pad use) and early, long-term intervention.

*Educators* reported that 73% of students had received some preparation before puberty, mainly through sex-education programs (62%). Topics most frequently addressed included private vs. public body parts (87.5%) and good/bad touch (71.9%), while masturbation (37.5%) and menstruation (43.8%) were less commonly covered. Preferred delivery formats were one-to-one sessions (48.6%) or blended with group work (43.2%). More than half (56.8%) supported beginning sex education before adolescence (Table 5).

**Table 5:** Topics and strategies used by educators

	Options	% reporting
Preparation/content area	Prepared students before puberty	73.0%
	Sex education programs	62.0%
	General topics	55.2%
	Private/public body parts	87.5%
	Good vs. bad touch	71.9%
	Physical changes	53.1%
	Masturbation	37.5%
	Menstruation	43.8%
Session’s format	One-to-one sessions	48.6%
	Blended (one-to-one + group)	43.2%
	Group sessions only	8.1%

*Parents* described varied strategies, including role modeling, social stories, activity scheduling, and sibling involvement, but also highlighted barriers, including communication difficulties, resistance, inconsistent school support, and financial costs.

All groups underscored the importance of early, individualized preparation. Yet, interventions remain fragmented, often reactive, and rarely embedded in a coordinated curriculum.

Resources and educator preparedness

*Specialists* pointed to systemic gaps: lack of trained sub-specialists, absence of comprehensive curricula, limited research on adolescence, and cultural taboos around discussing sexuality. Parents similarly described inadequate school programs and limited access to services, often resorting to online self-education. Financial constraints further restrict access to therapies.

*Educators* reported mixed access to resources: while 73% had some, more than half rated them only moderately effective. Most (65%) strongly endorsed the need for a specific ASD-focused curriculum. Although 69.8% felt capable of teaching sex education, 62.1% reported feeling embarrassed or uncomfortable, and 81.1% requested professional training. Collaboration with parents (73%) and professional visits (70.2%) were considered essential supports (Table 6). Correlation analysis reinforced these findings: teacher capability was positively associated with parental cooperation (r = 0.54, p = 0.001) and access to resources (r = 0.69, p < 0.001), while embarrassment correlated negatively with capability (r = -0.38, p = 0.021). Advocacy for curriculum necessity was most strongly predicted by parental cooperation (r = 0.86, p < 0.001) (Table 7).

**Table 6:** Resources and educator preparedness

Resource/preparedness item	Reporting
Access to resources	73.0%
Rated resources as moderately effective	56.7%
Strong need for a specific curriculum	65.0%
Felt capable of teaching sex ed	69.8%
Felt embarrassed/uncomfortable	62.1%
Desired professional training	81.1%
Cooperation with parents important	73.0%
Value professional visits	70.2%

**Table 7:** Correlations among teacher variables This statistical analysis reflects the relationship between variables with p < 0.05 (*), p ≤ 0.01 (**), and p < 0.001 (***).

Variable	1	2	3	4	5	6	7	8	9
1. Severity impact	1								
2. Sex education helpful	0.49**	1							
3. Resource efficiency	0.16	0.29	1						
4. Resource boundaries	0.18	0.31	0.77**	1					
5. Curriculum necessity	0.39*	0.72**	0.17	0.36	1				
6. Capability	0.28	0.58**	0.18	–0.10	0.56**	1			
7. Embarrassment	–0.08	–0.26	–0.08	–0.10	–0.42*	–0.38*	1		
8. Parental cooperation	0.41*	0.65**	0.15	0.33	0.86**	0.54**	–0.37	1	
9. Professional visits	0.05	0.06	0.46*	0.32	0.25	–0.01	0.03	0.34	1

Across stakeholders, persistent barriers included resource constraints, cultural discomfort, and insufficient training. Teacher confidence and advocacy depended strongly on collaboration and access to structured supports.

The Pearson correlation patterns reinforce the narrative findings;educators’ preparedness is closely tied to external support and internal confidence, both of which are shaped by structural and cultural dynamics. When parents are engaged and resources are accessible, teachers are more likely to feel capable and advocate for curriculum reform. Conversely, feelings of embarrassment, rooted in cultural discomfort, can undermine professional confidence and instructional willingness.

This multidimensional convergence between qualitative and quantitative strands strengthens the interpretive validity of the findings and highlights key levers for systemic improvement.

## Discussion

This study explored the challenges associated with puberty and sex education among adolescents with ASDin Lebanon through an exploratory sequential mixed-methods design. By integrating the perspectives of parents, special educators, and allied health professionals, the study identified five central domains: puberty-related changes and challenges; severity; gender-specific considerations; intervention practices; and resources and preparedness. The inclusion of both qualitative narratives and quantitative associations provided a nuanced understanding of how cultural, structural, and emotional factors intersect to shape experiences in this sensitive developmental stage.

Puberty-related changes and challenges

Findings confirmed that puberty intensifies difficulties in communication, boundary awareness, sensory regulation, and emotional control for adolescents with ASD. While such challenges have been reported globally [23,24],the Lebanese context adds distinctive cultural dimensions. Parents in this study emphasized hygiene management and menstruation-related difficulties, areas that are often underexplored in the international literature. Educators’ reports of high prevalence of boundary-crossing behaviors mirror findings elsewhere [25], but in Lebanon, these behaviors carry heightened stigma due to conservative cultural norms around sexuality. Parents and educators in Lebanon reported avoiding direct conversations about puberty, often due to embarrassment or fear of encouraging inappropriate behavior. This aligns with findings from similar sociocultural contexts [26]. The data underscore the need for culturally sensitive education that includes explicit guidance on hygiene, privacy, and emotional regulation, topics often under-addressed globally, especially in underserved or stigmatized populations.

Severity

The severity of ASD symptoms, especially language and communication limitations, emerged as a primary factor shaping both the challenges faced and the effectiveness of interventions. Consistent with Travers and Tincani (2010), the study found that adolescents with more severe or minimally verbal ASD presented greater support needs, while those with mild-to-moderate ASD were more responsive to structured sex education efforts [27]. Significantly, educators’ endorsement of curriculum reform was positively correlated with their experience of working with more severely affected adolescents. These findings suggest that the developmental and communicative profile of adolescents with ASD should inform intervention strategies globally and call for scalable, differentiated programs that accommodate a spectrum of needs.

Gender-specific considerations

A novel contribution of the study is the observation that gender plays a critical role in shaping puberty experiences and parental responses. Female adolescents with ASD were described as facing heightened sensory burdens during menstruation and hygiene management, often prompting proactive support from caregivers. In contrast, parents of boys typically adopted reactive strategies to address behaviors such as public masturbation. These culturally situated gender differences contrast with more uniform approaches reported in Western contexts [11]. The findings support calls for gender-sensitive curricula and interventions that are not only neurodevelopmentally appropriate but also responsive to sociocultural norms. Given that such gendered experiences are likely present in other conservative or low-resource contexts, this insight broadens the study’s relevance to international audiences.

Intervention practices

Across all groups, there was consensus on the importance of early, proactive, and individualized interventions. Educators emphasized basic sex-education topics such as body boundaries and good/bad touch, but were less likely to address masturbation or menstruation, reflecting cultural discomfort. This aligns with research from other Middle Eastern contexts where sexuality education remains fragmented and selective [28]. Specialists called for multidisciplinary collaboration, yet parents and educators highlighted its absence in practice. Quantitative findings showed that embarrassment was negatively correlated with teacher capability, indicating that cultural taboos directly inhibit professional confidence. This study, therefore, contributes new evidence from Lebanon that culture, specifically embarrassment, functions as a measurable barrier to effective sex-education delivery. However, the success of any intervention depends on the resources available and on educators' and families' preparedness. The final theme addressed these structural and contextual enablers.

Resources and preparedness

A shortage of structured resources and trained specialists was reported by all participant groups, echoing evidence from low- and middle-income countries more broadly [29]. While many teachers had some access to resources, they rated them as only moderately effective, and most expressed discomfort with delivery. Parents frequently resorted to self-education online, underscoring institutional gaps. Quantitative data demonstrated that teachers’ capability and curriculum advocacy were strongly associated with parental cooperation and resource access, while embarrassment consistently undermined both. This highlights a crucial point: teacher preparedness is less about individual competence and more about systemic support and collaborative networks. The Lebanese case adds to international literature by showing how cultural and structural barriers interact to limit the effectiveness of existing resources.

Integration and global implications

Taken together, the findings demonstrate that effective sex education for adolescents with ASD requires early, differentiated, and culturally anchored approaches. However, successful implementation depends on collaborative structures and professional support networks. Cultural stigma, manifested as embarrassment, is a measurable barrier to delivery, while family-school cooperation is a key enabler [8]. Although situated in Lebanon, these themes resonate across many low-resource and conservative settings.

This study contributes new insights by being one of the first mixed-methods investigations in the Arab region to explore puberty and sex education for adolescents with ASD. By triangulating the perspectives of parents, educators, and allied professionals, it sheds light on a neglected global health issue: the exclusion of neurodivergent youth from sexual education. The study’s identification of embarrassment as a quantifiable factor inhibiting teacher preparedness and advocacy is especially noteworthy, offering a direction for future research and intervention design.

By aligning with global health agendas such as the UN Sustainable Development Goals (SDG 3: Good Health and Well-being; SDG 4: Quality Education) and international guidance from WHO and UNESCO on inclusive sexuality education [30], the study reinforces the imperative to address sexual health inequities among marginalized youth. The results may inform policy discussions not only in Lebanon but also in other regions facing similar sociocultural and systemic constraints.

Implications for practice, policy, and research

The findings of this study carry significant implications for practice, policy, and research. In practice, teachers require structured training that addresses not only technical skills but also cultural taboos. At the same time, stronger parent-educator collaboration and clinicians' active involvement in multidisciplinary teams are essential for supporting individualized plans. At the policy level, there is a pressing need for educational authorities in Lebanon to invest in a culturally adapted, evidence-based sex education curriculum for ASD, complemented by continuous professional development for teachers and by adequate support for schools and associations to overcome financial and structural barriers. From a research perspective, future studies should evaluate culturally sensitive intervention models, compare proactive and reactive parental strategies, and examine gender-specific differences in preparation and outcomes. Longitudinal designs, in particular, would be valuable in clarifying how preparedness shapes adolescent adaptation over time.

Limitations

Several limitations must be acknowledged. The qualitative sample was modest, particularly the allied professional group (n = 3), which limits generalizability and saturation. Participant recruitment relied on snowball sampling, which may have introduced bias toward more engaged or better-resourced educators. While the teacher sample offered quantitative breadth, the analysis was exploratory and limited to correlations. The findings should thus be interpreted as hypothesis-generating rather than confirmatory. Lastly, the study was conducted exclusively in Lebanon, and while contextual insights are transferable, caution should be exercised in generalizing results to dissimilar cultural or systemic environments.

Future directions for research

Future studies should consider adopting a longitudinal design to examine the long-term effects of proactive sex education initiatives. It is also important to involve adolescents with ASD directly as participants, ensuring that their perspectives and experiences are represented. Further research could explore institutional practices and procedures across a range of therapeutic centers and educational institutions, providing insight into systemic strengths and gaps. Finally, expanding the scope of inquiry within the Arab world to include diverse cultural and socioeconomic contexts would contribute to a more comprehensive understanding of the challenges and opportunities in this field.

## Conclusions

This study highlights the multifaceted challenges adolescents with ASD face during puberty in Lebanon. Across interviews and the teacher survey, common concerns included emotional and sensory difficulties, hygiene and boundary-related issues, and sexual behaviors such as touching private parts (70.3%), hugging (67.6%), and masturbation (54.1%), with emotional dysregulation frequently linked to meltdowns (73%). Severity (especially limited communication) and gender shaped needs and experiences. Although 73% of educators reported providing puberty preparation, key topics such as masturbation (37.5%) and menstruation (43.8%) were less frequently addressed. Educator confidence was positively associated with parental cooperation (r = 0.54) and access to resources (r = 0.69) and negatively associated with embarrassment (r = -0.38). These findings underscore the need for structured, culturally appropriate, gender-sensitive, evidence-based curricula supported by parental collaboration and professional training to promote dignity, autonomy, and social inclusion.

## Appendices

Annex 1: survey to special educators

Greetings,

You are invited to participate in a research study at the University of Sciences and Arts in Lebanon entitled "Challenges of Puberty and Sex Education for Adolescents with Autism Spectrum Disorder: Perspectives of Parents and Specialists in the Lebanese Context in 2024", with the aim of understanding the challenges of adolescence with ASD from the perspective of special educators.

Participation in this study is entirely voluntary, and all responses will be kept confidential.

This survey will take approximately 5-6 minutes to complete.

We kindly seek your invaluable responses.

Section 1: Demographics

· Gender:

Male

Female

· Work region:

Beirut

Mount Lebanon

North Lebanon

South Lebanon

Baalbek-Hermel

Other: ______

· Years of experience in teaching students with ASD:

1-2 years

3-5 years

5-10 years

>10 years

· Age group of students with ASD taught:

<10 years

10- 17 years

>17 years

· Degree:

Bachelor

Masters

Teaching diploma

BT, TS

Other: ______

· Major:

Special education

Early childhood education

Arabic/English education

Math and sciences education

Other: ______

Section 2: Challenges During Puberty

1. Do you observe any challenges your students with ASD face during puberty?

Yes

No

2. What challenges do you think are mostly encountered during puberty?

Masturbation

Touching private parts

Hugs

Kisses

Aggression

Mood swings

Interest in the other sex

Getting in touch with the teacher

Pinching

Rubbing oneself with objects

Taking off clothes

Others ________

3. How do you observe adolescents with ASD coping with changes in routine and expectations during puberty?

Resistance and rigidity

Developing coping strategies

Increased anxiety

Regression

Escaping or avoidance

Seeking reassurance or clarification

Other: ______

4. In your experience, what specific sensory sensitivities tend to be most problematic for adolescents with ASD during puberty?

Hypersensitivity to sound (their sound or surrounding sounds)

Tactile hypersensitivity (change in skin texture, hair growth, hygiene...)

Visual sensitivity

Sensory overload (multiple sensory changes)

Proprioceptive and vestibular sensitivities (change of body size, disruption of body awareness)

Other: ______

5. What are the challenges related to communication and social skills development for adolescents with ASD during puberty?

Increased social complexity

Changes in social expectations

Difficulty with abstract language and humor

Self-awareness and self-advocacy (make decisions)

Executive function challenges (organizing)

Sensory sensitivities

Anxiety and social withdrawal

6. How do you rate the negative impact of puberty-related challenges on social interactions in your classroom?

1 - Strongly disagree

2

3

4

5 - Strongly agree

7. What are some common triggers for meltdowns or shutdowns in adolescents with ASD during puberty?

Sensory overload

Emotional regulation difficulties

Changes in routine

Unmet sensory or emotional needs

Social stressors

Transitions and unexpected changes

Communication difficulties

Other: ______

Section 3: Intervention

8. Did the students receive any type of preparation before reaching the age of puberty?

Yes

No

If yes, what were the techniques used for this preparation?

Sex education program

General topics

Other: ____________

9. If a sex education program was applied, which topics were handled?

Hygiene

Body awareness

Private and public body parts

Private and public behaviors

Masturbation

Menstruation (for girls)

Physical change

Good, bad touch

Other: ____________

10. In your opinion, what resources or support services would be most beneficial for adolescents with ASD navigating puberty?

Curriculum resources

Collaboration with professionals (multi-disciplinary team)

Parental involvement

Sensory tools and environmental accommodation

Ongoing training for special educators

Community partnership with local clinics or organizations

Other: ____________

11. How do you collaborate with parents or caregivers to address the challenges of puberty for adolescents with ASD outside of the school setting?

Open communication

Family-centered approach

Setting shared goals

Sharing strategies and techniques

Problem-solving together

Offering emotional support

Other: ____________

12. Do you think it is appropriate to take sex education in the form of?

One-to-one session

Group class

Mixed of both

13. Estimating the age needed by students to gain the necessary knowledge and skills regarding puberty, at what age do you think sex education should start?

Before adolescence phase

At the beginning of the adolescent phase

14. On a scale of 1 to 5, to which extreme did the severity of the autism case affect their ability to acquire sex education?

1 - Strongly disagree

2

3

4

5 - Strongly agree

15. Do you consider sex education helpful in overcoming the challenges of puberty?

1 - Strongly disagree

2

3

4

5 - Strongly agree

16. Regarding gender, do males and females differ in overcoming challenges of puberty when sex education is offered?

Yes

No

If yes, which of them is less capable?

Males

Females

Section 4: Resources of Interventions

17. Do you have access to the needed resources regarding sex education? (stories, videos, power points, curriculum)

Yes

No

18. Are the available resources efficient in teaching sex education?

1 - Strongly disagree

2

3

4

5 - Strongly agree

19. Do the available resources set clear boundaries about the appropriate teaching and managing behavior skills?

1 - Strongly disagree

2

3

4

5 - Strongly agree

20. What are the strategies used with students with ASD in teaching sex education?

Social stories

Animated videos

No animated videos

Simple instruction

One-to-one lesson

Visual strips

Life-sized dolls

Modelling

Role play

Other: _______

21. On a scale from 1 to 5, do you think that developing a specific curriculum of sex education for adolescents with ASD is necessary?

1 - Strongly disagree

2

3

4

5 - Strongly agree

Section 5: Concerning Teachers’ Readiness

22. Do you feel that you are capable of teaching sex education to adolescents with ASD?

1 - Strongly disagree

2

3

4

5 - Strongly agree

23. Do you feel embarrassed or uncomfortable when explaining sex education topics?

1 - Strongly disagree

2

3

4

5 - Strongly agree

24. Do you believe that cooperating with parents helps in reaching the goals of sex education?

1 - Strongly disagree

2

3

4

5 - Strongly agree

25. Do you think you may benefit from professionals’ visits, such as doctors, to your class?

1 - Strongly disagree

2

3

4

5 - Strongly agree

26. Do you think that having professional training may help in providing better quality of sex education?

Yes

No

Maybe

27. What are the topics that should be targeted in the training?

Common puberty-related challenges for adolescents with ASD (sensory, social, emotional)

Teaching sexuality and puberty education

Social skills instruction

Sensory needs and regulation

Personal hygiene and self-care

Crisis management training

Other: _______

Annex 2: semi-structured interview guide for autism specialists

Ø Target Participants

Autism specialists working with adolescents diagnosed with autism spectrum disorder (ASD), including board-certified behavior analysts (BCBA), occupational therapists (OT), and psychiatrists.

Ø Purpose

This interview guide was developed to explore professional perspectives on puberty-related challenges and sexual education among adolescents with ASD within the Lebanese context. The guide aims to capture clinical observations, intervention practices, family collaboration, and systemic gaps, while allowing discipline-specific probing based on the specialist’s professional role.

Ø Interview Characteristics

The interviews were semi-structured and open-ended. Each interview lasted approximately 40-60 minutes and was conducted either in person or online, depending on participant availability. Interviews were conducted in Arabic or English, at the participant's preference. All interviews were audio-recorded following informed consent. Each specialist participated in a single individual interview.

Ø Structure of the Interview Guide

A single core interview guide was administered to all autism specialists (BCBAs, OTs, and psychiatrists). During the interviews, role-specific probes were used to explore behavioral, functional, or medical aspects relevant to each specialty without altering the core questions. This approach was adopted to ensure consistency across specialist perspectives while allowing discipline-specific depth through targeted probing.

Core Interview Questions

What are the most commonly observed behavioral or emotional changes during adolescence among individuals with ASD?

In what situations do parents most commonly seek professional (behavioral, functional, or clinical) intervention during this stage?

What factors do you believe contribute to the emergence or escalation of these behaviors during puberty?

How do puberty-related challenges differ according to gender among adolescents with ASD?

Are there differences in behavioral or psychological challenges between adolescents with ASD and their neurotypical peers?

What strategies or interventions are used to help adolescents with ASD understand the physical and psychological changes associated with puberty, and how effective are these strategies in your experience?

To what extent does the severity of ASD influence the intensity of challenges and the type of interventions applied during adolescence?

From your professional perspective, how does a proactive (preventive) approach compare to a reactive approach in reducing puberty-related challenges?

What role do parents play in interventions during this stage, and what challenges arise in collaborating with families?

What resources are currently available for parents regarding puberty and sexual education for adolescents with ASD?

Role-Specific Probing

Ø Probes for BCBA and OT

These probes were used further to explore behavioral and functional dimensions of puberty-related challenges:

How are behavioral or functional skills addressed during puberty-related interventions?

What role do routines, task analysis, visual supports, or social stories play in intervention?

How is skill generalization supported across home, school, and community settings?

Ø Probes for a psychiatrist

These probes were used to explore medical and clinical considerations:

When should medical or pharmacological intervention be considered during adolescence?

How do psychiatric comorbidities influence puberty-related challenges in adolescents with ASD?

What guidance is typically provided to parents regarding medication management during this stage?

Appendix 3: semi-structured interview guide for parents of adolescents with ASD

Ø Target Participants

Parents or primary caregivers of adolescents diagnosed with ASD.

Ø Purpose

This interview guide aims to explore parents’ lived experiences of supporting their adolescents with ASD during puberty. The questions focus on emotional, behavioral, educational, and practical challenges, access to services, and parental coping strategies within the Lebanese context.

Ø Interview Characteristics

The interviews were semi-structured and open-ended, lasting approximately 40-60 minutes. Interviews were conducted in Arabic or English, according to the participant's preference, and were audio-recorded after informed consent was obtained.

Ø Interview Questions

Have you noticed any changes in your child’s behavior or emotions during puberty, and to what extent did you feel prepared for this stage?

What specific challenges has puberty posed for your child with ASD?

How did your family manage these challenges, including the roles of siblings and parents?

What challenges did you face in helping your child understand and cope with bodily changes during puberty?

What services or strategies were used during this phase, and which were most effective in supporting your child?

Did financial factors affect your ability to access appropriate services or support?

How did you address issues related to self-care and hygiene during this stage, if applicable?

Were there any resources or support networks that were particularly helpful for you and your child?

Did schools or centers provide sexual education for your child or guidance for you as parents?

What advice would you give to other parents whose children with ASD are approaching or experiencing puberty? In your opinion, is early preparation beneficial?
